# Hypoxia-Induced Ferroptosis Resistance Drives Orbital Fibrosis in Thyroid Eye Disease

**DOI:** 10.1167/iovs.67.4.15

**Published:** 2026-04-08

**Authors:** Chengzhen Gong, Shu Liu, Jing Zhao, Jingya Zhu, Chenqi Xing, Kelin Li, Chifeng Xie, Naijia Wu, Rongxin Chen

**Affiliations:** 1 State Key Laboratory of Ophthalmology, Zhongshan Ophthalmic Center, Sun Yat-Sen University, Guangdong Provincial Key Laboratory of Ophthalmology and Visual Science, Guangzhou, China; 2 State Key Laboratory of Oncology in South China, Guangdong Provincial Clinical Research Center for Cancer, Sun Yat-Sen University Cancer Center, Guangzhou, China; 3 School of Medicine, Sun Yat-Sen University, Guangzhou, China

**Keywords:** hypoxia, ferroptosis resistance, fibrosis, lactate, thyroid eye disease

## Abstract

**Purpose:**

To determine whether hypoxia promotes a ferroptosis-resistant phenotype in patient-derived thyroid eye disease (TED) orbital fibroblasts (OFs), and whether hypoxia-associated metabolic changes (lactate) relate to profibrotic activation readouts, as well as to test whether modulating ferroptosis alters these readouts.

**Methods:**

Primary OFs from TED patients and controls were exposed to graded hypoxia (21% to 1% O₂). Ferroptosis sensitivity was assessed by reactive oxygen species, mitochondrial membrane potential, viability, and SLC7A11/GPX4/ACSL4. The profibrotic phenotype was assessed by COL1A1/α-SMA/fibronectin, immunofluorescence, and scratch assays. We perturbed metabolism with exogenous lactate and 2-deoxy-D-glucose, induced ferroptosis with RSL3, and inhibited ferroptosis with liproxstatin-1.

**Results:**

TED fibroblasts exhibited baseline ferroptosis resistance, marked by higher GPX4 and SLC7A11 and lower ACSL4 than controls, which intensified under hypoxia. Hypoxia alone was sufficient to increase fibrotic marker expression and further augmented TGF-β–evoked fibrotic responses. Under hypoxia, RSL3 at 50 to 100 nM reduced profibrotic marker expression in TED OFs, an effect lost at 150 nM where viability declined; liproxstatin-1 attenuated the RSL3-associated changes. Hypoxia increased intracellular lactate; lactate alone upregulated GPX4 and promoted fibrotic activation, whereas 2-deoxy-D-glucose decreased lactate, restored RSL3 sensitivity, and diminished fibrotic readouts.

**Conclusions:**

Our data suggest that hypoxia-associated lactate accumulation contributes to GPX4-linked ferroptosis resistance and amplifies profibrotic activation in TED OFs. Mechanism-guided intervention combining metabolic inhibition with calibrated ferroptosis induction warrants further investigation as a rational strategy to restrain fibrotic remodeling.

Hypoxia is a recognized driver of tissue fibrosis in multiple pathological contexts, including hepatic and pulmonary fibrosis, primarily through fibroblast activation.^1^^–^^5^ Thyroid eye disease (TED), an autoimmune orbital disorder, is characterized by progressive fibrosis mediated by orbital fibroblasts (OFs).^6^^–^^8^ The dysregulation of OFs in TED leads to pathologic overproduction of collagen and fibronectin, thereby driving extracellular matrix remodeling.^9^^–^^11^ Accumulating evidence supports the presence of a hypoxic microenvironment in TED orbits. In a previous study, we observed increased hypoxia-inducible factor-1 alpha (HIF-1α) levels in orbital tissues from patients with TED compared with controls (Supplementary Fig. S1). This clinically grounded evidence, together with prior reports showing that HIF-1α signaling in TED OFs modulates inflammatory and fibrotic responses,^12^^–^^17^ prompted us to investigate hypoxia-driven mechanisms in TED. Recent findings further corroborate this link. A machine learning–assisted study integrating bioinformatic and experimental wet-lab data identified hypoxia-related pathways as significantly associated with TED pathogenesis.^18^

Despite these indications, current in vitro TED fibrosis models predominantly rely on exogenous TGF-β1 under normoxia, failing to recapitulate the hypoxic orbital milieu.^19^^,^^20^ Although reproducible TSHR-based immunization animal models have been established and have advanced mechanistic studies of TED, they may show variability in orbital manifestations and do not fully reflect the heterogeneity and chronic course of human TED. Moreover, increasing ethical and practical incentives to decrease animal use further motivate the development of complementary, well-controlled nonanimal platforms.^21^^–^^25^ This study directly addresses the functional impact of hypoxia on TED OFs, establishing a novel, hypoxia-driven fibrotic model.

Hypoxia has a complex impact on ferroptosis, a form of regulated cell death characterized by intracellular reactive oxygen species (ROS) accumulation, dysregulated iron metabolism, and lipid peroxidation.^26^ Under hypoxia, ferroptosis regulation is highly context dependent and reflects the balance between iron-driven lipid peroxidation stress and adaptive antioxidant defenses. In some settings, hypoxia exacerbates ferroptosis in RPE cells by enhancing the Fenton reaction,^27^ leading to further tissue damage. Similarly, a lnc-HZ06/HIF-1α-SUMO feedback loop under hypoxia triggers ferroptosis in trophoblasts, contributing to miscarriage.^28^ In contrast, other cell types adapt to hypoxia by strengthening ferroptosis-suppressing programs. For example, hypoxia suppresses ferroptosis in ovarian cancer cells and promotes growth and metastasis via metabolic reprogramming (including SLC2A12 upregulation).^29^ Hypoxia can also upregulate SLC7A11, thereby enhancing resistance to ferroptosis in glioma through the PI3K/AKT/HIF-1α axis.^30^ Recent evidence has suggested that HIF-1α modulates ferroptosis in muscle by promoting ferritinophagy and lactate production.^31^

Importantly, emerging studies indicate that TED is associated with ferroptosis resistance. TED OFs have been shown to display significantly reduced erastin-induced lipid peroxidation, quantified as a lower oxidized-to-reduced fluorescence intensity ratio using a lipid peroxidation sensor, compared with control OFs, consistent with increased tolerance to ferroptotic stress.^32^ This study explores the relationship between hypoxia-induced ferroptosis resistance and profibrotic processes in TED OFs, hypothesizing that manipulating ferroptosis may influence fibrotic processes.

We observed that enhanced glycolysis under hypoxic conditions leads to significant lactate accumulation. Emerging evidence suggests that lactate functions not only as a byproduct of glycolysis,^33^ but also as a key signaling molecule regulating both ferroptosis and fibrosis through lactylation-mediated modifications of the epigenome and protein functions.^34^^–^^36^ In TED, ferroptosis resistance has been partially attributed to increased glycolytic flux, and insulin-like growth factor-1 receptor (IGF-1R)—a major therapeutic target in TED—has been reported to promote glycolysis; conversely, IGF-1R blockade can enhance ferroptosis sensitivity.^32^ Moreover, in other fibrotic contexts, lactate and TGF-β1 form a positive feedback loop that sustains collagen synthesis, cell proliferation, and migration, thus driving fibrotic progression.^37^^,^^38^ Lactate also indirectly contributes to fibrosis by impairing mitochondrial function and inducing oxidative or reductive stress.^39^^,^^40^ Collectively, hypoxia-driven lactate accumulation has been implicated in promoting ferroptosis resistance and informing profibrotic programs via lactylation-dependent and TGF-β–associated mechanisms.^32^^,^^34^^–^^38^ Whether a similar hypoxia–lactate–ferroptosis/fibrosis coupling operates in TED OFs remains unclear. This study further explored how the hypoxia–lactate–ferroptosis axis influences TED pathogenesis, particularly in promoting fibrotic phenotypes through the suppression of ferroptosis and activation of fibrosis-related signaling pathways.

## Methods

### Tissue Samples and Cell Isolation

Human orbital connective tissues were surgically obtained from TED patients undergoing orbital decompression (retrobulbar intraconal fat, *n* = 11) and control subjects undergoing enucleation or cosmetic procedures (retrobulbar intraconal fat, *n* = 4; eyelid fat, *n* = 2).^41^^–^^43^ For TED patients, comprehensive clinical parameters were prospectively documented at enrollment, including age, gender, smoking history, Clinical Activity Score (CAS), disease activity status (active = CAS ≥ 3; inactive = CAS < 3), and disease duration. Critically, all TED patients maintained stable euthyroid status at the time of surgery (confirmed by serum thyroid-stimulating hormone, free T3, and free T4 levels), and none had received prior biologic therapy for TED. All participants met the core inclusion criterion of no immunosuppressant or glucocorticoid therapy within 3 months before tissue sampling (Table 1). Full demographic and clinical characteristics for all participants are provided in Supplementary Table S1. Informed consent was obtained from all subjects after a detailed explanation of the study procedures and potential implications. The research was conducted in compliance with the ethical approval from Zhongshan Ophthalmic Center (2016KYPJ028). The human tissue experiments complied with the guidelines of the ARVO Best Practices for Using Human Eye Tissue in Research (Nov2021).

**Table 1.** Demographic and Clinical Characteristics of Tissue Donors

	TED Group (*n* = 11)	Control Group (*n* = 6)	*P* Value
Age (years)	56.3 ± 7.8	57.3 ± 2.3	0.721
Gender (female/male)	5/6	2/4	>0.999
CAS score^*^	2.4 ± 1.6	—	NA
Smoking history (yes/no)	5/6	3/3	>0.999
Retrobulbar intraconal fat/eyelid fat	11/0	4/2	

* CAS range is 1–7.

The collected tissues were processed for both paraffin embedding and primary fibroblast isolation. For cell culture, tissue specimens were carefully minced into approximately 1-mm³ fragments and explanted onto culture dishes containing Dulbecco's Modified Eagle Medium (Gibco, Waltham, MA, USA) supplemented with 20% fetal bovine serum (ExCell Bio, URU) and 1% penicillin-streptomycin (Gibco). The cultures were maintained at 37°C in a humidified 5% CO₂ atmosphere. For each experiment, a minimum of three independent primary OF lines derived from different donors were used as biological replicates. Each cell line was assayed in technical triplicates, and the mean of the triplicates was calculated to represent that biological replicate for statistical analysis. The exact number of independent lines (*n*) used for each experiment is indicated in the respective figure legends. Cells between passages 3 to 8 were used.^22^^,^^44^^,^^45^

### Hypoxia Treatment

OFs were exposed to graded hypoxic conditions (10%, 5%, 3%, or 1% O₂) using a controlled hypoxia chamber (Anoxomat MARK III, Norwood, MA, USA) for 24 hours. Normoxic controls (21% O₂) were maintained in standard incubator conditions.^46^^–^^48^

### Lentiviral Overexpression of HIF-1α in OFs

To achieve HIF-1α overexpression, TED OFs were transduced with a lentiviral vector carrying the HIF-1α gene. The HIF-1α expression plasmid construct (in a lentiviral vector containing GFP:T2A:Puro selection markers) was obtained from VectorBuilder Biosciences (Guangzhou, China). For lentivirus production, 293T cells at 90% confluence were transfected with this construct. Viral supernatants were harvested at 24, 36, and 48 hours post transfection, filtered through a 0.45 µm membrane, concentrated using Amicon filters (Millipore, Burlington, MA, USA), and stored at −80°C. For transduction, OFs were seeded at a density of 5 × 10⁵ cells/mL in expansion medium and incubated with the harvested lentivirus, supplemented with 10 µg/mL polybrene to enhance infection efficiency. After 18 hours, the virus-containing medium was replaced with fresh culture medium. Transduced OFs were used for subsequent experiments 24 hours post infection.

### Western Blotting

Cells were lysed in RIPA buffer (Beyotime, Sanghai, China) and quantified via BCA assay. Proteins (30 µg/lane) were separated by SDS-PAGE and transferred to polyvinylidene fluoride membranes. After blocking, membranes were incubated overnight at 4°C with primary antibodies: anti-GPX4 (1:2000, Abmart, Shanghai, China), anti-SLC7A11 (1:2000, Abmart), anti-ACSL4 (1:2000, Abmart), anti-fibronectin (1:1000, Abcam, Cambridge, UK), anti-COL1A1 (1:1000, Abcam), and anti–α-SMA (1:1000, Abcam). Horseradish peroxidase–conjugated secondary antibodies (1:5000, Cell Signaling Technology, Danvers, MA USA) were applied for 1 hour. Signals were detected using ECL (Millipore, Burlington, MA, USA) and analyzed with ImageJ.

### RT-qPCR

Total RNA was extracted using Trizol reagent (AG, Shanghai, China) and reverse transcribed into cDNA with the PrimeScript RT reagent kit (Vazyme, Jiangsu, China). Gene-specific primer sequences are detailed in Table 2. qRT-PCR was performed using SYBR Green (Vazyme) on a real-time PCR machine (Roche LightCycler 480, Basel, Switzerland), with β-actin used as the reference gene because its expression is stable under hypoxic conditions, unlike GAPDH, which is known to be regulated by hypoxia.^49^ The expression of target genes was calculated using the 2^−ΔΔCt^ method.

**Table 2.** Gene-Specific Primer Sequences for qPCR Analysis

Gene	Primer Sequence (5′→3′)
*GPX4*	F: GAGGCAAGACCGAAGTAAACTAC
	R: CCGAACTGGTTACACGGGAA
*SLC7A11*	F: TCTCCAAAGGAGGTTACCTGC
	R: AGACTCCCCTCAGTAAAGTGAC
*ACSL4*	F: CATCCCTGGAGCAGATACTCT
	R: TCACTTAGGATTTCCCTGGTCC
*HIF-1α*	F: GCCACTTCTTCTGTAAGTCTGTGGG
	R: TCAACTGGTCTCAAGTCAGTG
*Fibronectin*	F: ACAAGCATGTCTCTCTGCCAA
	R: GCAATGTGCAGCCCTCATTT
*TIMP1*	F: CATCACTACCTGCAGTTTTGTG
	R: TGGATAAACAGGGAAACACTGT
*ITGB1*	F: CTGTGATGCCTTACATTAGCAC
	R: ATCCAAATTTCCAGATATGCGC
*β-Actin*	F: CATGTACGTTGCTATCCAGGC
	R: CTCCTTAATGTCACGCACGAT

### Immunohistochemistry (IHC) and Immunofluorescence (IF) Staining

Paraffin sections (5 µm) underwent antigen retrieval (citrate buffer, 95°C, 15 minutes). After blocking (5% BSA, 1 hour), samples were incubated overnight at 4°C with primary antibodies: anti-GPX4 (1:100), anti-SLC7A11 (1:100), anti–α-SMA (1:100), and anti-COL1A1 (1:100). For IHC, horseradish peroxidase–conjugated secondary antibody (1:500, Abcam) and DAB (Proteintech, Wuhan City, China) were used. For IF, Alexa Fluor-594 secondaries (1:1000, Abcam) were applied (1 hour, dark). Nuclei were counterstained with hematoxylin (IHC) or DAPI (IF). Slides were imaged using Nikon Eclipse microscope for IHC and LSM 880 focal microscope (Zeiss, Jena, Germany) for IF.

For each independent specimen, three or more fields were captured under identical imaging settings and quantified using ImageJ (NIH). IHC staining was quantified as positive area fraction (% area), and IF signals were quantified from the same set of images; values were averaged across fields to obtain one value per specimen for statistical analysis.

### CCK-8 Assay

Cells were seeded in 96-well plates and treated under experimental conditions. After incubation, 10 µL CCK-8 reagent (Gotion High-Tech, Hefei City, China) was added per well and incubated at 37°C for 2 hours. Absorbance at 450 nm was measured using a microplate reader (BioTek, Winooski, VT, USA). Cell viability was calculated as a percentage relative to the time-matched, oxygen-matched untreated control (i.e., normoxia samples normalized to normoxic controls and hypoxia samples normalized to hypoxic controls).

### Scratch Wound Healing Assay

Confluent TED OF monolayers in six-well plates were scratched with a 200-µL sterile pipette tip. After PBS washes, wounds were imaged at 0 (baseline), 24, and 48 hours using an Eclipse Ti2 microscope (Nikon, Tokyo, Japan). Hypoxic (1% O₂) and normoxic groups were maintained in 1% fetal bovine serum medium. Wound areas were quantified in ImageJ by measuring the residual gap width relative to baseline, from which the cell migration rate was calculated.

### Oxidative Stress Detection

Intracellular ROS levels were quantified using the dichloro-dihydro-fluorescein diacetate probe from the Reactive Oxygen Species Assay Kit (Beyotime). Cultured cells on six-well plates were incubated with 10 µM dichloro-dihydro-fluorescein diacetate diluted in serum-free medium at 37°C for 20 minutes under light-protected conditions. Following triple washing with warm PBS (Gibco), fluorescence imaging was immediately conducted on an Eclipse Ti2 inverted fluorescence microscope (Nikon).

### Mitochondrial Membrane Potential (ΔΨm) Assessment

ΔΨm was assessed using the JC-1 Assay Kit (Beyotime) following the manufacturer's specifications. Positive control samples were treated with 50 µM carbonyl cyanide m-chlorophenylhydrazone for 20 minutes at 37°C. Cultured fibroblasts grown on glass-bottom dishes (NEST, Wuxi, China) were incubated with 5 µg/mL JC-1 staining solution at 37°C for 20 minutes protected from light. After triple washing with prewarmed JC-1 buffer, live cell imaging was immediately performed using a LSM 880 focal microscope (Zeiss). JC-1 aggregates (red; high ΔΨm) and monomers (green; low ΔΨm) were acquired under identical imaging settings across groups. Where quantification was required, ΔΨm was expressed as the green/red fluorescence intensity ratio measured from multiple nonoverlapping fields per sample using ImageJ (NIH) (normalized to the time-matched, oxygen-matched untreated control).

### Electron Microscopy

For electron microscopy, cells were fixed in 2.5% glutaraldehyde, post fixed in 1% osmium tetroxide, and dehydrated through a graded ethanol series. Ultra-thin sections were stained with uranyl acetate and lead citrate and analyzed under a transmission electron microscope (JEOL, Tokyo, Japan).

### Lactate Assay and Glycolysis Inhibition

Intracellular lactate levels were quantified using a lactate assay kit (Solarbio, Beijing, China) following the manufacturer's protocol. TED OFs were treated with exogenous sodium L-lactate (10–20 mM; TargetMol, Wellesley Hills, MA, USA) for 24 hours before analysis. Glycolytic flux was pharmacologically inhibited using 10 mM 2-deoxy-D-glucose (2-DG; TargetMol).

### Statistical Analysis

All experimental procedures were independently replicated a minimum of three times, and data are presented as the mean ± SEM. Statistical analysis was performed using Prism 8 (GraphPad, Boston, MA, USA). Normality of data distribution was assessed using the Shapiro–Wilk test, and homogeneity of variances was evaluated using the Brown–Forsythe test; all datasets met the assumptions for parametric testing. Differences between groups were assessed using the Student *t*-test or one-way ANOVA, followed by Tukey's post hoc test for multiple comparisons. For experiments involving two factors (e.g., oxygen tension and drug concentration), separate one-way ANOVAs were performed within each factor level to address the specific hypotheses. *P* values of <0.05 were considered statistically significant.

## Results

### GPX4/SLC7A11-Mediated Ferroptosis Resistance Exists in TED

**Figure 1.**
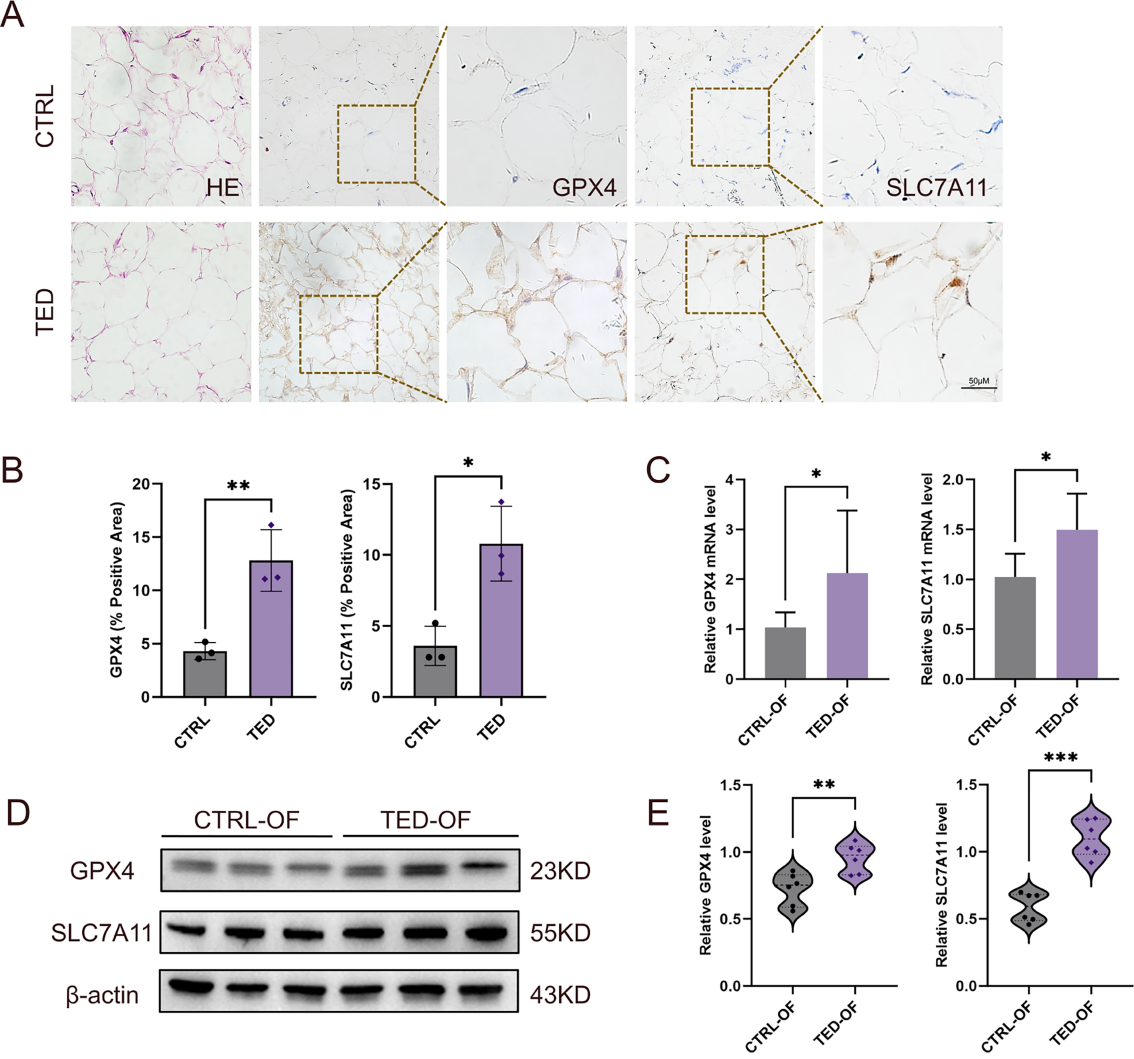
**GPX4-SLC7A11-mediated ferroptosis resistance exists in TED.** (**A**) Representative IHC staining of GPX4 and SLC7A11 in orbital tissues from TED patients (*n* = 3) and controls (*n* = 3), showing elevated protein expression in TED. *Scale bar*, 50 µm. (**B**) Quantification of IHC staining in (**A**) based on ImageJ analysis (positive area fraction, % area); values represent the average of three or more fields per specimen. (**C**) Relative mRNA expression of GPX4 and SLC7A11 validated by qRT-PCR in TED OFs (*n* = 6) vs. CTRL OFs (*n* = 6). (**D**, **E**) Western blot analysis showing increased protein expression of GPX4 and SLC7A11 in primary OFs derived from TED OFs (*n* = 6) compared with CTRL OFs (*n* = 6). β-Actin served as a loading control. Data are expressed as mean ± SEM. *P < 0.05, **P < 0.01, ***P < 0.001.

To investigate ferroptosis resistance, we isolated primary OFs from TED patients (TED OFs) and healthy controls (CTRL OFs) and assessed the relevant mRNA and protein expression in both cultured cells and orbital tissue specimens. Western blot analysis revealed significantly elevated protein levels of key ferroptosis suppressors GPX4 (*P <* 0.01) and SLC7A11 (*P* *<* 0.001) in TED OF cultures compared with CTRL OFs (Figs. 1A, 1B), with concordant upregulation at the mRNA level confirmed by qRT-PCR (Fig. 1C). IHC staining of orbital tissue sections further corroborated these findings, showing intensified GPX4 and SLC7A11 immunoreactivity in TED connective tissues (Fig. 1D). Additionally, enhanced expression of NRF2—a transcriptional activator of antioxidant genes including SLC7A11—was observed in TED OFs (Supplementary Fig. S2), suggesting potential upstream reinforcement of ferroptosis defense. Collectively, these alterations in TED-derived fibroblasts and tissues demonstrate constitutive upregulation of ferroptosis defense mechanisms, providing direct evidence of ferroptosis resistance in TED pathogenesis.

### Hypoxia-Mediated Ferroptosis Resistance in TED OFs via the SLC7A11/GPX4 Axis

**Figure 2.**
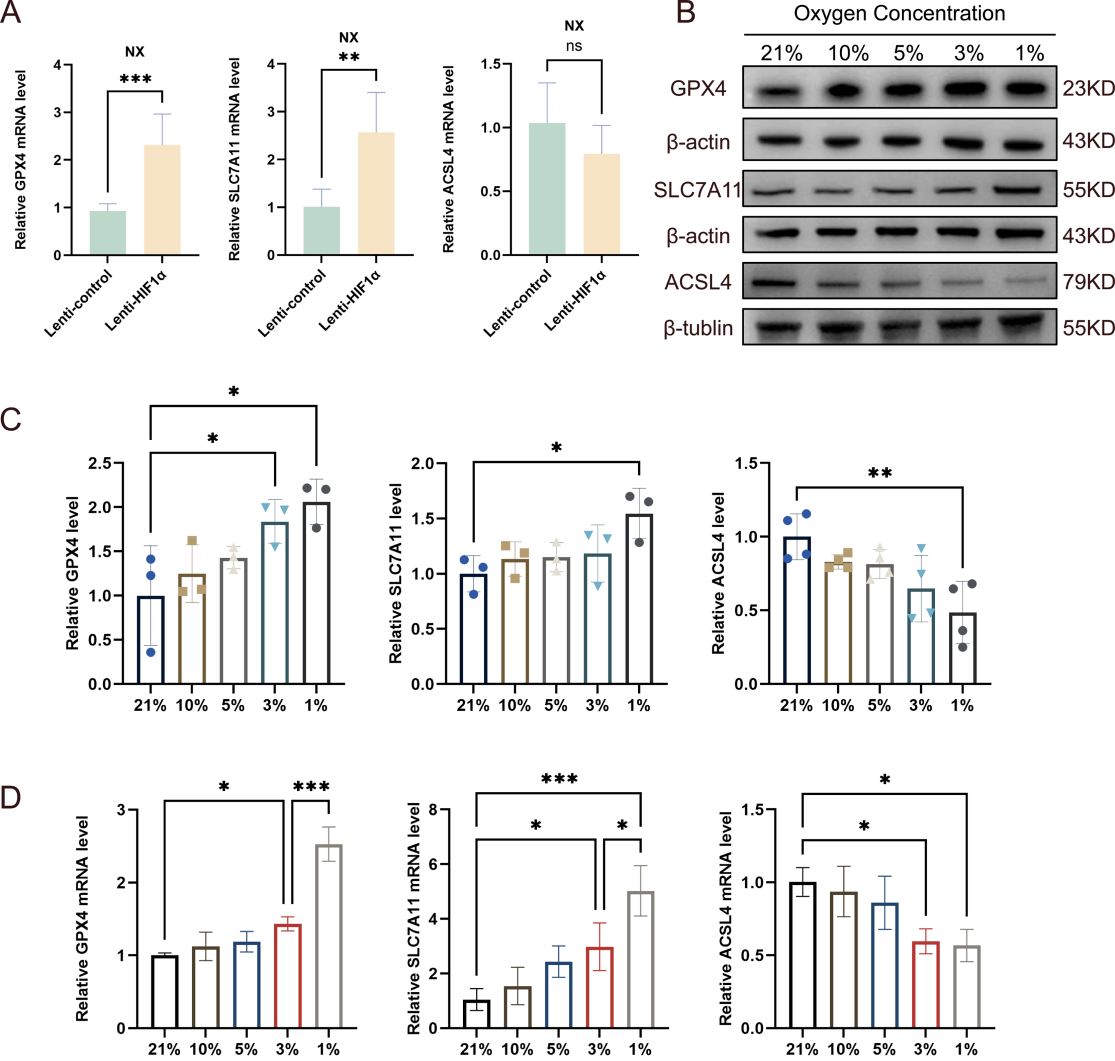
**Hypoxia mediates ferroptosis resistance in TED OFs through the SLC7A11/GPX4 axis.** (**A**) qRT-PCR analysis of SLC7A11, GPX4, and ACSL4 mRNA expression in TED OFs (*n* = 3) overexpressing HIF-1α under normoxic conditions (21% O₂), with β-actin as an internal control. (**B**, **C**) Western blot analysis of SLC7A11, GPX4, and ACSL4 protein levels in TED OFs (*n* ≥ 3) exposed to graded hypoxia (21% to 1% O₂). β-Actin and β-tubulin served as a loading control. (**D**) qRT-PCR quantification confirms progressive hypoxia-induced increase in SLC7A11 and GPX4 mRNA and reduction of ACSL4 expression in TED OFs (*n* ≥ 3) exposed to graded hypoxia (21% to 1% O₂), with β-actin as an internal control. Data are expressed as mean ± SEM. *P < 0.05, **P < 0.01, ***P < 0.001.

We investigated the causal link between hypoxia and ferroptosis resistance in TED. First we overexpressed HIF-1α in TED OFs using lentiviral vectors under normoxic conditions. qRT-PCR analysis at 24 hours post transfection showed increased mRNA expression of the ferroptosis-suppressing genes SLC7A11 and GPX4, and ACSL4 mRNA showed a modest downward trend without reaching statistical significance (Fig. 2A). Further exposing TED OFs to graded hypoxia revealed oxygen-dependent regulation. Compared with normoxic controls, a progressive reduction in oxygen concentration induced corresponding molecular changes. Western blot (Figs. 2B, 2C) and qRT-PCR (Fig. 2D) analyses consistently showed gradual upregulation of SLC7A11 and GPX4 expression coupled with declining ACSL4 levels across decreasing oxygen tensions. Maximal induction of ferroptosis resistance occurred at 1% O_2_, where TED OFs exhibited peak expression of protective factors SLC7A11 and GPX4, concurrent with maximal suppression of ACSL4. These findings establish hypoxia as a pivotal regulator of antiferroptotic reprogramming in TED OFs.

### Hypoxia Exposure Divergently Modulates Ferroptosis Sensitivity in TED OFs and CTRL OFs

**Figure 3.**
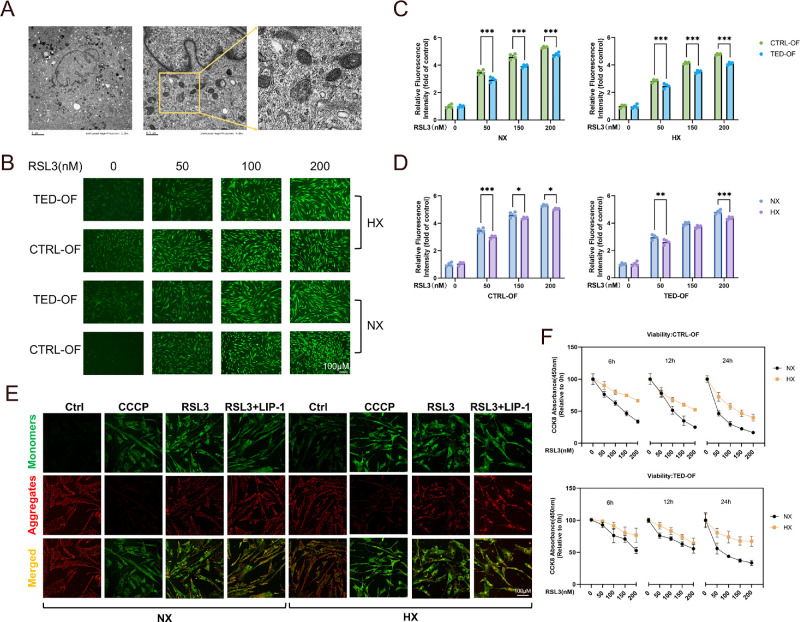
**Differential modulation of ferroptosis sensitivity by hypoxia in TED OFs and CTRL OFs.** (**A**) Electron microscopy images of mitochondria in RSL3-treated TED OFs under hypoxia. (**B**) Representative fluorescent microscopy images showing reduced ROS accumulation in TED OFs (*n* = 4) compared with CTRL OFs (*n* = 4) upon RSL3 exposure under hypoxic and normoxic conditions. *Scale bar*, 100 µm. (**C**) Analysis of relative fluorescence of comparison of the response to RSL3 treatment between CTRL OFs and TED OFs under normoxic and HX (1% O_2_) conditions. (**D**) Analysis of relative fluorescence of comparison of the response to RSL3 treatment between normoxic and HX in CTRL OFs and TED OFs. (**E**) JC-1 staining to evaluate ΔΨm in TED OFs treated with RSL3 under normoxia and hypoxia. LIP-1 (10 µM) was used as a ferroptosis inhibitor control. Carbonyl cyanide m-chlorophenylhydrazone served as a mitochondrial depolarization control. *Red*, JC-1 aggregates (high ΔΨm); *green*, JC-1 monomers (low ΔΨm). *Scale bar*, 100 µm. (**F**) Cell viability assays showing metabolic activity in TED OFs (*n* = 6) and CTRL OFs (*n* = 6). *Scale bar*, 100 µm. Data are expressed as mean ± SEM. *P < 0.05, **P < 0.01, ***P < 0.001.

Having established hypoxia-induced upregulation of the SLC7A11/GPX4 axis at molecular levels, we next evaluated its functional consequences on mitochondrial homeostasis and cellular resilience to ferroptotic stress. Electron microscopy confirmed characteristic ferroptotic morphology in RSL3-treated TED OFs, including shrunken mitochondria with condensed cristae and ruptured outer membranes, validating ferroptosis induction (Fig. 3A). Subsequent ROS detection (Fig. 3B) revealed concentration-dependent ROS accumulation in response to RSL3 across all conditions, but TED OFs consistently exhibited attenuated ROS generation compared with CTRL OFs under both normoxic and hypoxic environments (Figs. 3C, 3D).

This oxidative stress resistance was further corroborated by JC-1 ΔΨm assessment (Fig. 3E). Carbonyl cyanide m-chlorophenylhydrazone (positive control) induced rapid ΔΨm depolarization with accelerated kinetics under hypoxia. RSL3 treatment triggered ΔΨm depolarization in all groups, and no consistent oxygen-dependent difference was apparent from the JC-1 staining patterns. Liproxstatin-1 (LIP-1) partially mitigated RSL3-induced ΔΨm disruption, with comparable efficacy under both oxygen tensions.

Cell viability profiling demonstrated superior survival in hypoxic conditions for both fibroblast types, with TED OFs maintaining significantly higher metabolic activity than CTRL OFs throughout the 24-hour time course (Fig. 3F).

### Hypoxia Triggers Profibrotic Activation in TED OFs

**Figure 4.**
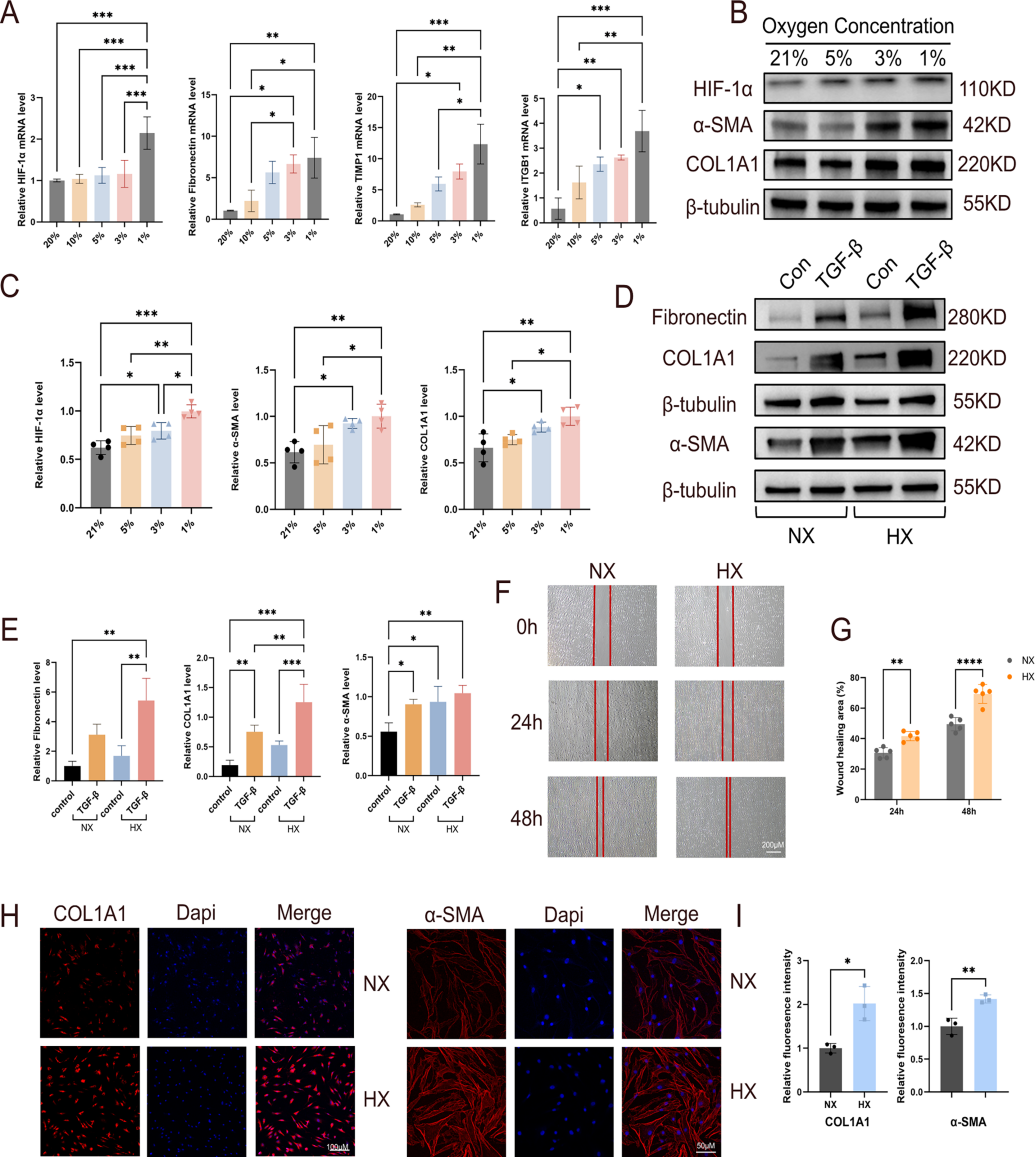
**Hypoxia induces fibrotic activation in TED OFs.** (**A**) qRT-PCR analysis of fibrosis-related genes (HIF-1α, fibronectin, TIMP1, ITGB1) in TED OFs (*n* = 4) exposed to graded hypoxia (21% to 1% O₂), with β-actin as an internal control. (**B**, **C**) Western blot analysis of HIF-1α and fibrotic markers COL1A1 and α-SMA in TED OFs (*n* = 4) exposed to graded hypoxia (21% to 1% O₂). β-Tubulin was used as a loading control. (**D**, **E**) Western blot analysis and quantification of COL1A1, α-SMA, and fibronectin in TED OFs (*n* = 4) under the indicated conditions (Normoxia, normoxia + TGF-β1; Hypoxia, hypoxia + TGF-β1). β-Tubulin was used as a loading control. (**F**, **G**) Scratch assay illustrating enhanced cell motility in TED OFs (*n* = 5) under hypoxic conditions. Time points: 0, 24, and 48 hours. (**H**, **I**) IF staining showing COL1A1 and α-SMA deposition in TED OFs (*n* = 3) exposed to 1% O_2_. *Scale bar*, 100 µm for COL1A1, 50 µm for α-SMA. Data are expressed as mean ± SEM. *P < 0.05, **P < 0.01, ***P < 0.001, ****P < 0.0001.

After 24 hours of exposure of TED OFs to graded hypoxia, we observed an oxygen-dependent fibrotic activation: decreasing oxygen tension (21% to 1% O₂) was associated with coordinated increases in molecular and functional fibrosis-related readouts. qRT-PCR analysis revealed significantly increased mRNA expression of HIF-1α, fibronectin, TIMP1, and ITGB1, showing inverse correlation with oxygen concentrations (Fig. 4A). Consistent with these transcript-level changes, Western blot analysis performed at the same time point confirmed HIF-1α protein accumulation under graded hypoxia, accompanied by increased expression of fibrotic hallmark proteins, including COL1A1 and α-SMA, with the strongest induction observed under 1% O₂ (Figs. 4B, 4C). We further compared the fibrotic effects of hypoxia with the canonical profibrotic cytokine TGF-β1. Although TGF-β1 (10 ng/mL)^19^^,^^22^ treatment robustly induced fibrotic activation, hypoxia alone, in the absence of exogenous TGF-β1, also significantly upregulated fibrotic markers (Figs. 4D, 4E). Notably, the combination of hypoxia and TGF-β1 resulted in the highest expression levels of COL1A1 and fibronectin, suggesting that the hypoxic microenvironment may cooperate with or potentiate TGF-β1 signaling to exacerbate fibrosis marker levels in TED OFs.

Functional validation through scratch assays showed accelerated wound closure under hypoxia, demonstrating enhanced OF motility (Figs. 4F, 4G). IF confirmed intense α-SMA filament organization and COL1A1 deposition in 1% O_2_ versus normoxic controls, quantifying extracellular matrix accumulation (Figs. 4H, 4I). Collectively, these data support a hypoxia-driven in vitro system that captures key fibrotic activation readouts in TED OFs without exogenous TGF-β1 stimulation, providing a controllable platform to interrogate hypoxia-associated mechanisms in OFs. The synergistic effect observed with combined hypoxia and TGF-β1 highlights a potentially critical pathological amplification loop in TED, warranting future investigation.

### RSL3-Induced Ferroptosis Attenuates Hypoxia-Driven Fibrotic Activation in TED OFs

**Figure 5.**
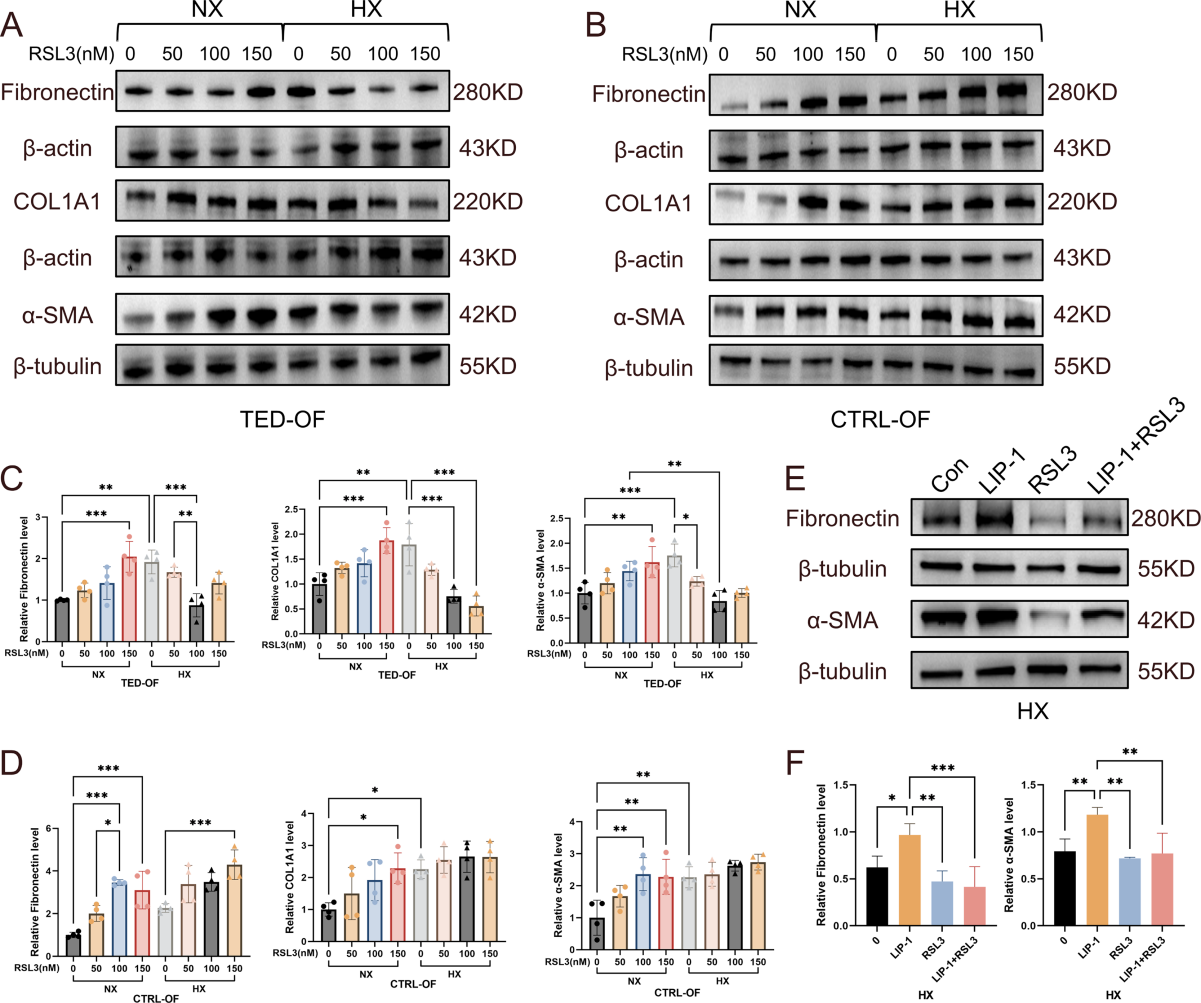
**Ferroptosis induction by RSL3 attenuates hypoxia-driven fibrotic activation in TED OFs.** (**A**, **B**) Western blot analysis of COL1A1, α-SMA, fibronectin, and β-actin protein levels in TED OFs (*n* = 4) and CTRL OFs (*n* = 4) treated with RSL3 under normoxic and hypoxic conditions. (**C**, **D**) Quantification of Western blot analysis. (**E**) Pharmacological rescue experiments under hypoxia (1% O₂). Western blot analysis of fibrotic markers in TED OFs (*n* = 4) treated with the ferroptosis inhibitor LIP-1 (5 µM) alone, or in combination with RSL3 (100 nM). (**F**) Quantification of Western blot analysis. Data are expressed as mean ± SEM. *P < 0.05, **P < 0.01, ***P < 0.001.

Having established that hypoxia independently drives both ferroptosis resistance and fibrotic activation in TED OFs, we next interrogated their functional interplay by challenging fibroblasts with ferroptosis inducer RSL3 under differential oxygen tensions. Western blot analysis revealed oxygen-contextualized fibrotic responses to ferroptotic stress. Under normoxic conditions (21% O₂), RSL3 treatment dose dependently (0–150 nM) increased fibrotic protein expression in both CTRL OFs and TED OFs, evidenced by progressive increases in COL1A1, α-SMA, and fibronectin across RSL3 concentrations (Fig. 5A, 5B). In contrast, hypoxic exposure (1% O₂) induced divergent fibrotic responses: CTRL OFs maintained RSL3 dose-dependent profibrotic activation, whereas TED OFs exhibited a paradoxical reduction in fibrotic markers (COL1A1/α-SMA/fibronectin) with increasing RSL3 concentrations. Quantification of fibrotic markers revealed concentration-dependent inflection points in hypoxic TED OFs (Figs. 5C, 5D). Specifically, fibronectin and α-SMA were reduced at 50 nM and reached their lowest levels at 100 nM, followed by a rebound at 150 nM. Thus, in hypoxic TED OFs, an observed in vitro concentration range (50–100 nM) of RSL3 was associated with attenuation of fibrotic marker expression, whereas a higher concentration (150 nM) was associated with a loss of this effect and marker rebound. The loss of an antifibrotic effect at 150 nM was accompanied by reduced cell viability (Fig. 3F), indicating that the fibrotic marker rebound may be attributable to cytotoxicity rather than a direct biological effect. Statistical re-analysis directly comparing 1% O_2_ and normoxic conditions at each RSL3 concentration confirmed that the attenuation of fibrotic markers was specifically significant under hypoxia (Supplementary Fig. S3).

To directly test the causal role of ferroptosis in this process, we used the ferroptosis inhibitor LIP-1 (10 µM).^50^ Critically, inhibition of ferroptosis by LIP-1 under hypoxia significantly increased the expression of fibrotic markers in TED OFs (Figs. 5E, 5F). This hypoxia-driven fibrotic activation was effectively reversed by cotreatment with 100 nM RSL3, confirming that the induction of ferroptosis is sufficient to counteract the profibrotic trajectory.

### Lactate Links Hypoxia to Fibrotic Activation and Ferroptosis Resistance

**Figure 6.**
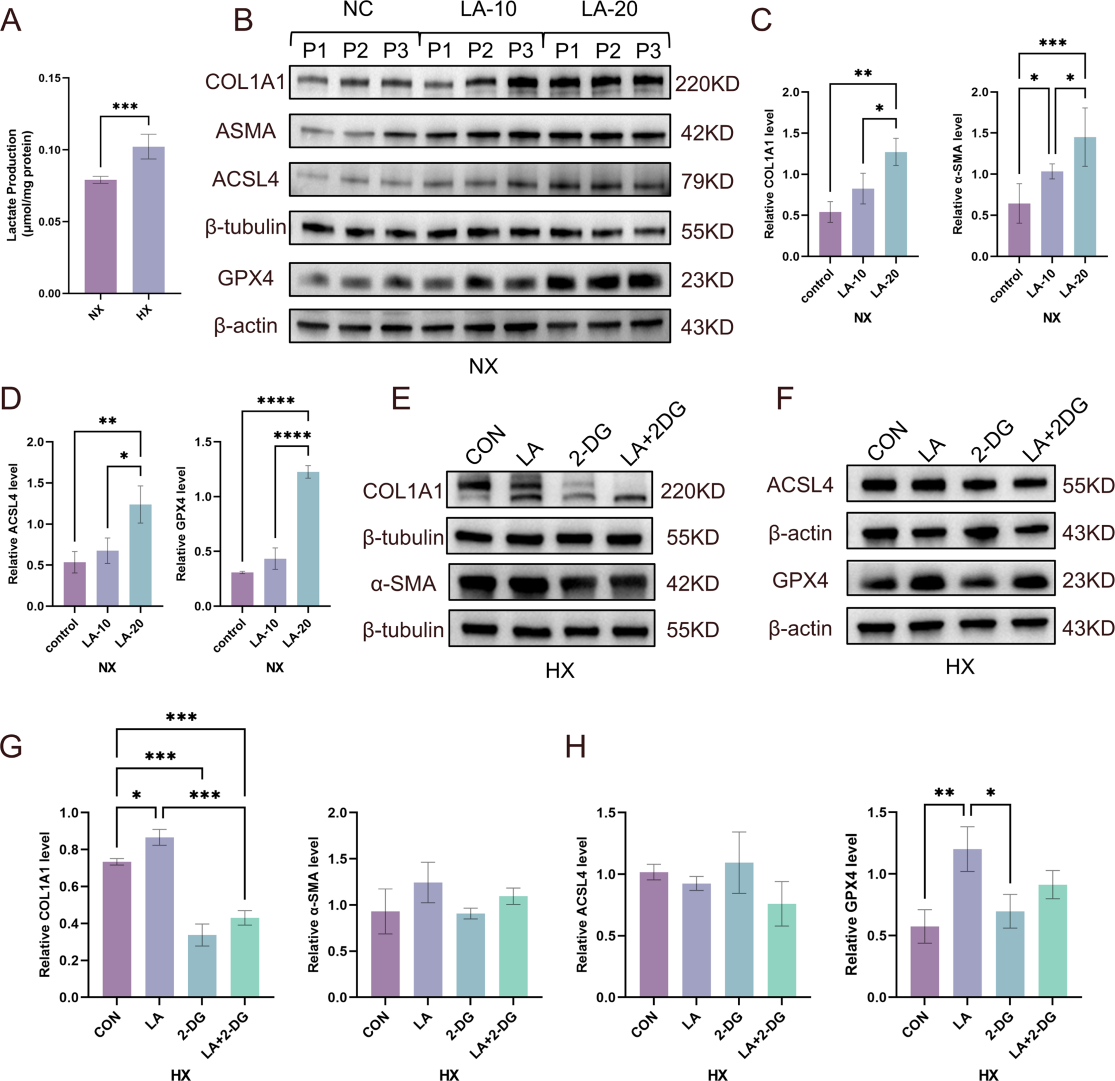
**Lactate amplifies hypoxia-induced fibrotic activation and GPX4-mediated ferroptosis resistance in TED OFs.** (**A**) Intracellular lactate quantification demonstrates increased lactate accumulation in TED OFs (*n* = 6) under hypoxia (HX). (**B**–**D**) Western blot analysis showing the dose-dependent effects of exogenous lactate (10–20 mM) on fibrotic markers (α-SMA, COL1A1) and ferroptosis related protein (GPX4, SLC7A11, and ACSL4) (*n* = 3; P, patient). (**E**, **G**) Western blot analysis demonstrating the effects of lactate on fibrotic markers (α-SMA, COL1A1) in TED OFs (*n* = 4) under hypoxic conditions. (**F**, **H**) Western blot analysis showing the effects of lactate on GPX4 and ACSL4 expression in TED OFs (*n* = 4) under hypoxic conditions, with β-actin and β-tubulin as an internal control. Data are expressed as mean ± SEM. *P < 0.05, **P < 0.01, ***P < 0.001.

The quantification of intracellular lactate confirmed significant accumulation in hypoxic TED OFs versus normoxic controls (Fig. 6A). Under normoxic conditions, exogenous lactate supplementation (10–20 mM)^51^^–^^53^ dose dependently upregulated the profibrotic markers α-SMA and COL1A1 (Figs. 6B, 6C). Western blot and qRT-PCR analyses collectively demonstrated that exogenous lactate supplementation under normoxia induced significant GPX4 upregulation at both transcriptional and translational levels, conferring ferroptosis resistance in TED OFs (Fig. 6D; Supplementary Fig. S4). ACSL4 showed a modest upward trend that did not attain statistical significance, suggesting a potential compensatory response. Functionally, lactate pretreatment markedly attenuated RSL3-induced ROS generation (Supplementary Fig. S5).

In hypoxic TED OFs, lactate accumulation coincided with enhanced fibrotic activation, and lactate supplementation further increased α-SMA and COL1A1 levels (Figs. 6E, 6G). Notably, treatment with the glycolytic inhibitor 2-DG attenuated these hypoxia-associated fibrotic responses, coinciding with reduced lactate levels. In parallel, hypoxia-associated ferroptosis resistance was accompanied by increased GPX4 and reduced ACSL4 levels (Figs. 6F, 6H), and 2-DG cotreatment attenuated these changes an effect that occurred alongside reduced lactate and partially restored ferroptotic sensitivity. Collectively, these findings support lactate as a hypoxia-linked metabolic mediator that contributes to profibrotic activation and is associated with GPX4-related ferroptosis resistance in TED OFs.

## Discussion

Our findings suggest that hypoxia is associated with fibrotic activation in TED OFs and is accompanied by a ferroptosis-resistant phenotype characterized by increased GPX4/SLC7A11 and metabolic changes including lactate accumulation. Under our experimental conditions, these hypoxia-associated profibrotic readouts were observed without adding exogenous TGF-β1, and exogenous TGF-β1 further amplified the response. In our in vitro system, pharmacological ferroptosis induction attenuated selected hypoxia-associated fibrotic readouts within a narrow concentration range, providing a mechanistic rationale for further preclinical evaluation.

Whereas prior in vitro TED models have relied primarily on exogenous TGF-β, our hypoxia-based paradigm better reflects an in-situ stressor and exposes a dose-dependent ferroptosis range that is not readily captured in TGF-β–only systems. In TED-derived fibroblasts, RSL3 at 50 to 100 nM suppressed hypoxia-driven fibrotic activation, whereas higher doses abrogated this benefit, indicating a biphasic response and the need for precise dosing. Whether a similar range exists in vivo, and whether it can be exploited safely, will require validation in appropriate animal models and/or ex vivo orbital tissue or organoid systems. We, therefore, view these findings as hypothesis generating, particularly for disease stages in which extraocular muscle swelling and orbital fat expansion increase orbital soft tissue volume and orbital space conflict/venous congestion, potentially compromising orbital perfusion and contributing to a hypoxic orbital microenvironment.^54^^–^^57^

The dose-dependent profibrotic effect of RSL3 under normoxia, while seemingly paradoxical, may reflect sublethal stress-induced compensatory signaling—a phenomenon increasingly recognized in redox biology.^58^ Sublethal ferroptotic stress can activate prosurvival pathways (e.g., MAPK/nuclear factor-κB) and TGF-β signaling, promoting matrix production rather than cell death.^59^ This interpretation aligns with the “ferroptosis range” concept: the cellular redox set point determines whether ferroptosis modulation achieves therapeutic elimination or triggers adaptive profibrotic responses. Under hypoxia, elevated GPX4/SLC7A11 shifts this threshold, enabling effective antifibrotic activity at comparable RSL3 concentrations.

**Figure 7.**
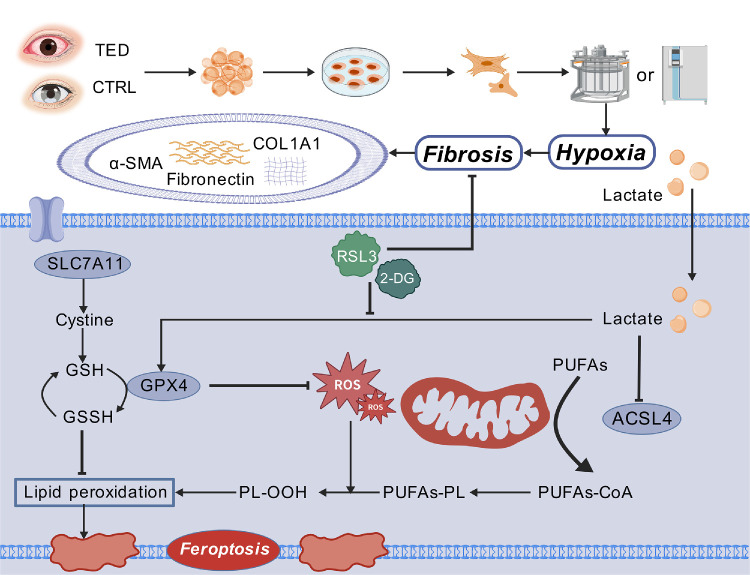
**Proposed mechanistic model of the hypoxia-lactate-ferroptosis resistance axis driving orbital fibrosis in TED.** OFs derived from TED patients exhibit enhanced fibrotic activation under hypoxia, characterized by increased α-SMA, COL1A1, and fibronectin expression. Hypoxia also promotes glycolytic reprogramming with lactate accumulation, which contributes to ferroptosis resistance by favoring the SLC7A11-GSH-GPX4 antioxidant axis and limiting lipid peroxidation/ROS-driven ferroptotic cell death. Lactate-associated metabolic alterations are linked to ACSL4 and PUFA metabolism, thereby influencing susceptibility to ferroptosis. Pharmacological interventions used in this study (e.g., RSL3 and 2-DG) are indicated. The schematic summarizes observed associations from our in vitro data; intermediate mechanisms represent potential links warranting future investigation rather than demonstrated direct causality. Created with BioGDP.com.

Mechanistically, lactate is a key amplifier of hypoxia-induced ferroptosis resistance and fibrotic activation. Hypoxia-driven glycolysis increased intracellular lactate, which upregulated GPX4 and SLC7A11 while suppressing ACSL4, thereby limiting lipid peroxidation and promoting matrix deposition. Exogenous lactate partially recapitulated hypoxic signaling, whereas glycolytic blockade with 2-DG decreased lactate, restored RSL3 sensitivity, and attenuated fibrotic activation, indicating a reversible, metabolism-dependent node upstream of ferroptosis resistance (Fig. 7). These findings, consistent with emerging literature on lactate's epigenetic and metabolic roles in fibrotic activation and ferroptosis,^60^^–^^64^ define a tractable therapeutic axis centered on lactate. Such an approach could complement current treatments targeting IGF-1R and inflammatory pathways.^65^^,^^66^

In the context of therapeutic implications, our work suggests that modulating the hypoxia–lactate–ferroptosis resistance axis may offer dual benefits: suppression of fibrotic activation and restoration of susceptibility to regulated cell death, while addressing the core fibrotic pathology more directly. Moreover, the establishment of a hypoxia-driven, TGF-β–independent fibrosis model offers a robust platform for high-fidelity drug screening and mechanistic studies.

Limitations include reliance on an in vitro hypoxia model, which requires validation in vivo or in ex vivo organoids to confirm translational relevance.^44^ Accordingly, any therapeutic implications of targeting ferroptosis in TED should be considered preliminary until validated in appropriate in vivo or ex vivo models. Moreover, our hypoxia experiments focused on a 24-hour exposure range; future work will include time-course analyses to better define the temporal dynamics of hypoxia-driven fibrotic activation. In addition, control adipose tissues were collected mainly from orbital fat and partly from eyelid fat pads obtained during cosmetic blepharoplasty; although these depots are anatomically related, minor site-dependent variability should be considered.^67^ Furthermore, although RSL3 was used as a prototypical ferroptosis inducer, the safety and efficacy of ferroptosis-targeting drugs in orbital tissues require further investigation. Future studies may also explore additional regulators of lactate metabolism, such as monocarboxylate transporter and histone lactylation,^68^ to dissect the broader regulatory network.

## Conclusions

Our data indicate that hypoxia-associated lactate accumulation and a ferroptosis-resistant phenotype coincide with enhanced profibrotic activation readouts in patient-derived TED OFs. The dose-dependent effects observed with ferroptosis modulation identify a tractable mechanistic relationship that warrants validation in ex vivo/in vivo models and cautious preclinical exploration. These findings advance our understanding of TED pathogenesis and motivate future studies aimed at validating and translating these mechanisms in orbital tissues.

## Supplementary Material

Supplement 1
