# Supplementary material for: Hypoxia-Induced Ferroptosis Resistance Drives Orbital Fibrosis in Thyroid Eye Disease
Source: Invest Ophthalmol Vis Sci. 2026 Apr 8;67(4):15. doi: 10.1167/iovs.67.4.15 (PMC13069343; doi:10.1167/iovs.67.4.15)
Supplement: Supplement 1 [file iovs-67-4-15_s001.pdf]

Supplementary Table: Clinical Characteristics of Patients

Patients	Gender (Famle/Male)	Age (Years)	Smoking History (Yes or No)	CAS Score	Activity Status	TED Duration (Months)
TED						
1	Famale	60	No	4	active	5
2	Male	52	Yes	1	inactive	4
3	Famale	48	No	1	inactive	10
4	Famale	66	No	5	active	6
5	Male	57	Yes	4	active	4
6	Male	43	Yes	2	inactive	13
7	Male	55	Yes	2	inactive	10
8	Male	69	No	1	inactive	7
9	Famale	52	No	1	inactive	12
10	Male	61	Yes	1	inactive	6
11	Famale	56	No	4	active	8
Control						
1	Male	53	Yes	/	/	/
2	Male	57	No	/	/	/
3	Male	57	Yes	/	/	/
4	Male	58	Yes	/	/	/
5	Famale	61	No	/	/	/
6	Famale	58	No	/	/	/

*TED activity status (CAS ≥ 3 = active, CAS < 3 = inactive).

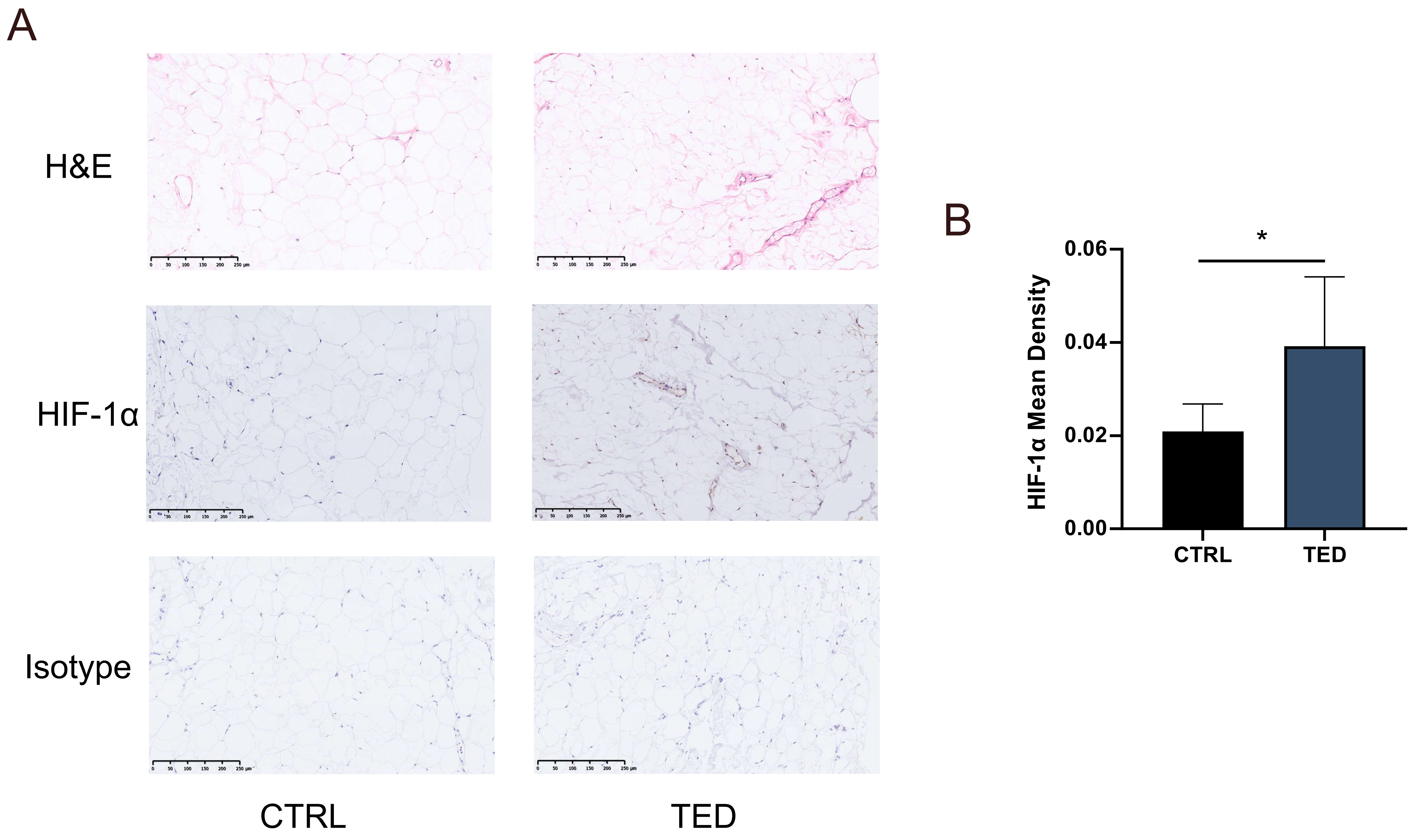

Supplementary Figure 1: HIF-1α is elevated in orbital connective tissues from patients with TED. (A) Representative hematoxylin and eosin (H&E) staining and immunohistochemistry (IHC) for HIF-1α on paraffin-embedded orbital connective tissue sections from control subjects and TED patients; an isotype-matched control is shown in parallel. Positive HIF-1α staining is visualized as yellowish-brown (DAB). Scale bars, 250 μm. (B) The mean density was used for the semi-quantitative analysis of IHC (TED, n = 6; CTRL, n = 6). Data are presented as mean ± SEM. Statistical significance was assessed using a two-tailed unpaired Student's t-test. *P < 0.05.

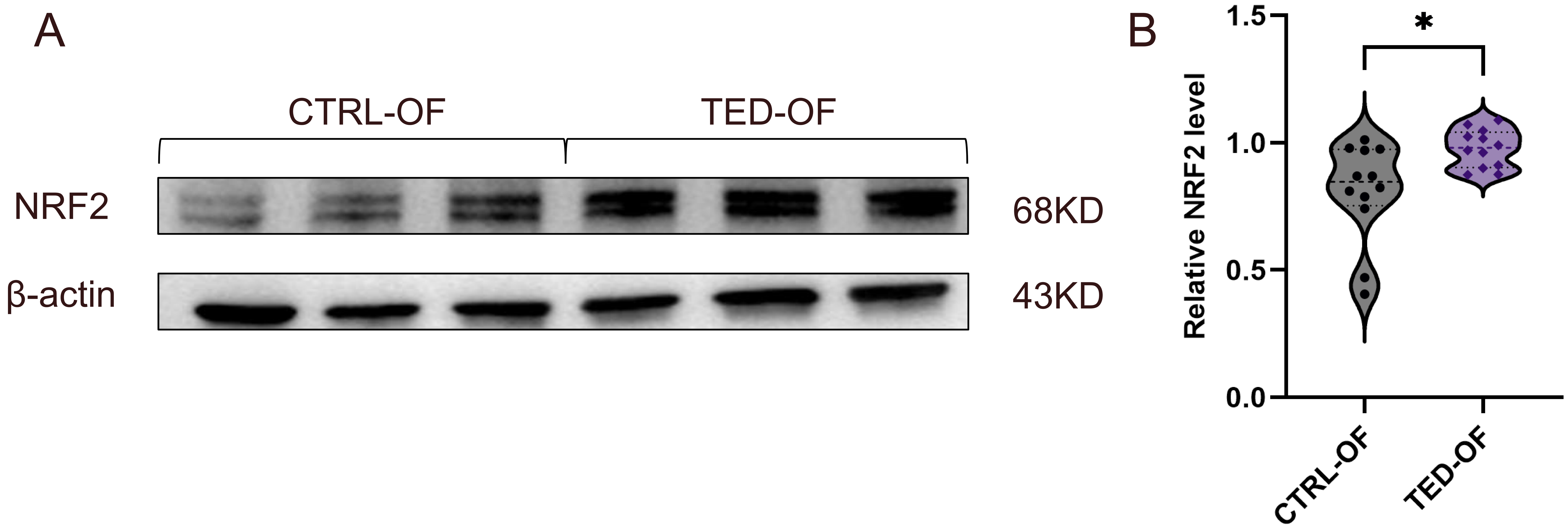

Supplementary Figure 2: NRF2 expression in TED-OFs and CTRL-OFs. Western blot analysis showing NRF2 protein levels in TED-OFs (n = 6) and CTRL-OFs (n = 6). β-actin was used as a loading control. Data are expressed as mean ± SEM. * P < 0.05.

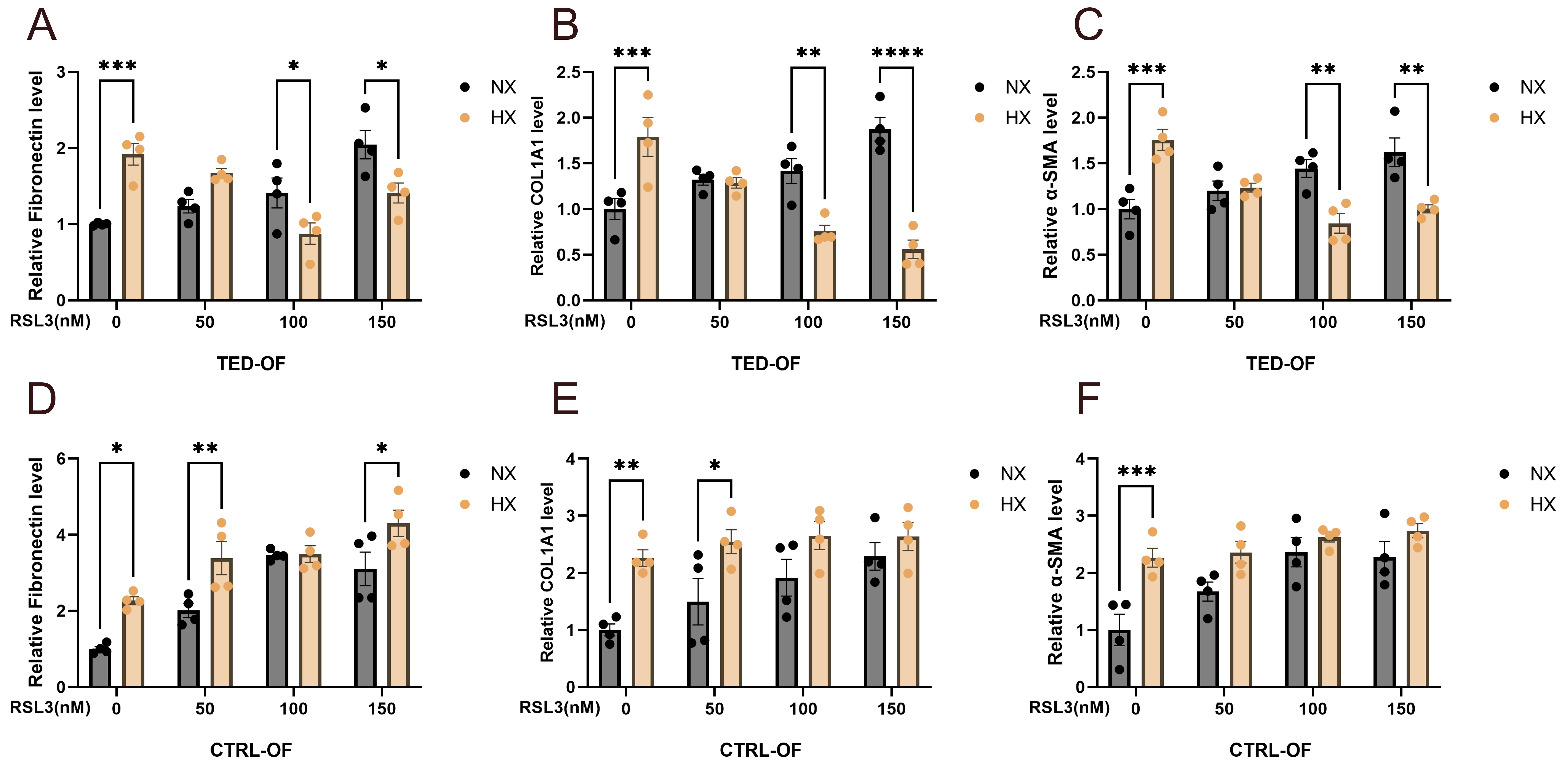

Supplementary Figure 3: Quantitative comparison of fibrotic marker expression in TED-OFs under normoxia (NX) versus hypoxia (HX) across increasing RSL3 concentrations. This figure provides an alternative visualization of the data presented in Fig. 5C and D, focusing on the direct statistical comparison between normoxia (NX) and hypoxia (HX) at each RSL3 concentrations. TED-Ofs (n = 4) were exposed to 1% O_2 (HX) or 21% O_2 (NX) and treated with RSL3 (0-150 nM) for 24 hours. Protein levels of (A) COL1A1, (B) α-SMA, and (C) Fibronectin were quantified by Western blot and normalized to β-actin or β-tubulin. Statistical comparisons were performed between NX and HX groups at each RSL3 concentration using unpaired Student's t-test. Data are expressed as mean ± SEM. * P < 0.05, ** P < 0.01, *** P < 0.001.

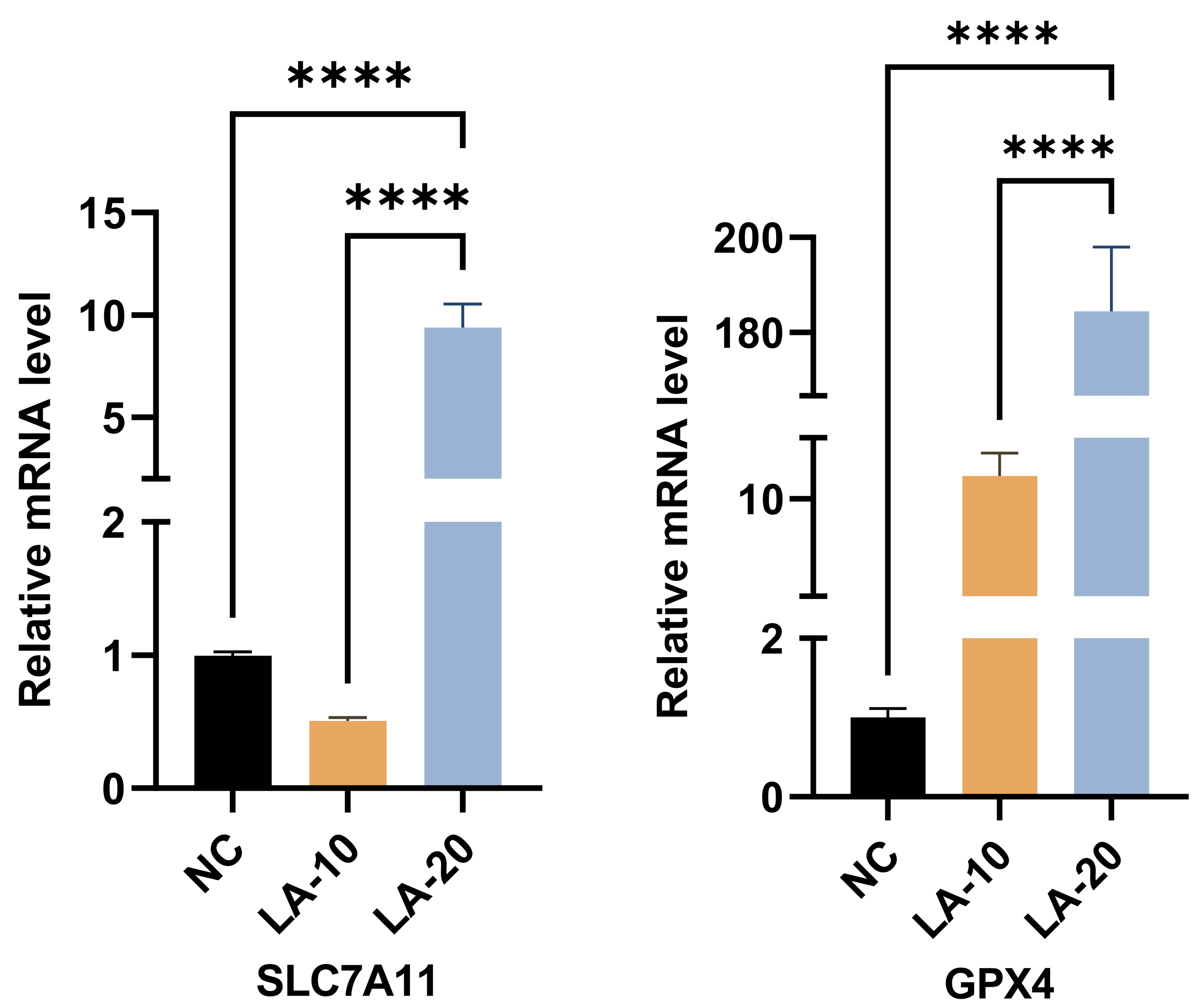

Supplementary Figure 4: Effect of exogenous lactate on SLC7A11 and GPX4 expression in TED-OFs. qRT-PCR analysis of SLC7A11 and GPX4 mRNA expression in TED-OFs treated with exogenous lactate (10-20mM) under normoxia, compared to control (NC), with β-actin as an internal control. Data are expressed as mean ± SEM. **** $P < 0.0001$.

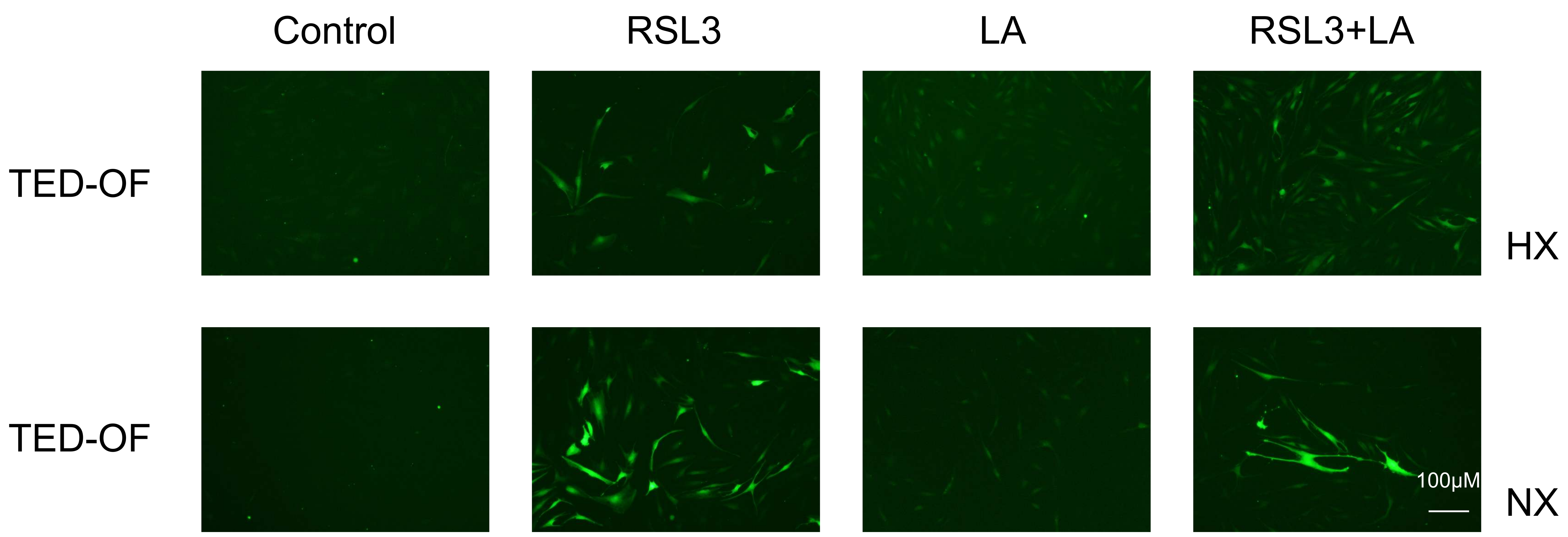

Supplementary Figure 5. Lactate enhances ferroptosis resistance in TED-OFs under hypoxia. Representative fluorescent microscopy images showing ROS accumulation in TED-OFs treated with RSL3, exogenous lactate (LA), and a combination of both (RSL3+LA) under hypoxic (HX) and normoxic (NX) conditions. Scale bar, 100 μm.
